# The neonatal gut microbiome in health and disease

**DOI:** 10.1080/19490976.2025.2457499

**Published:** 2025-01-27

**Authors:** Geoffrey A. Preidis

**Affiliations:** Division of Gastroenterology, Hepatology & Nutrition, Department of Pediatrics, Baylor College of Medicine and Texas Children’s Hospital, Houston, TX, USA; USDA/ARS Children’s Nutrition Research Center, Baylor College of Medicine, Houston, TX, USA

## Introduction

In the 18 y since the launch of the international Human Microbiome Project,^1,2^ our understanding of the complex relationships between gut microbes and their human hosts has increased substantially. These relationships are particularly dynamic during the first year of life, when events occurring during critical developmental windows may have life-long impacts on human health. In parallel, clinical care of the neonate (an infant <4 weeks old) has evolved rapidly. Infants born extremely preterm (<28 weeks of gestation) or with extremely low birth weight (ELBW, <1,000 g) now represent a patient population that did not exist for most of the history of medicine. Opportunities abound to leverage advances in microbiome science to improve survival, developmental trajectories, and long-term outcomes for vulnerable children.

**Figure 1.**
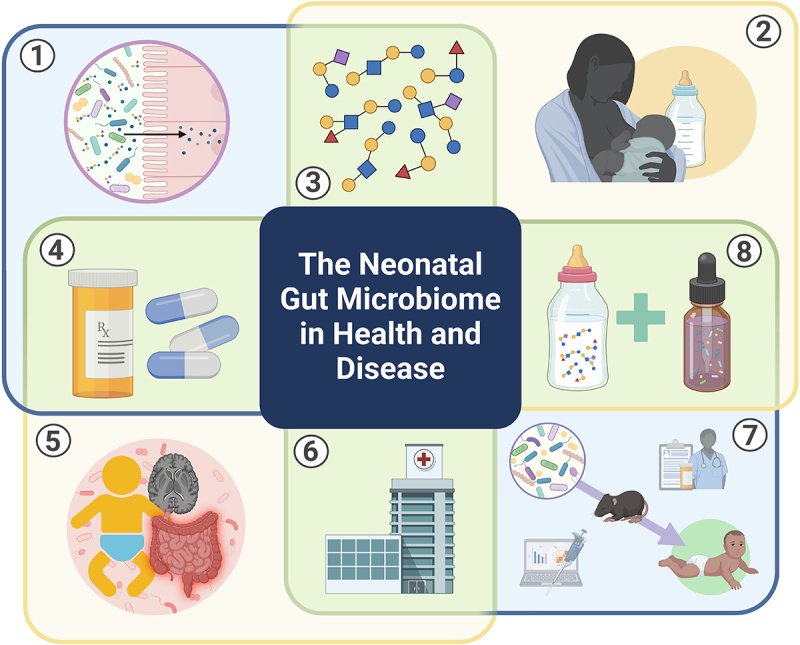
Schematic of eight topics explored in the special focus issue: 1) basic concepts in neonatal gut microbial colonization and functional maturation; 2) dietary influences on the neonatal gut microbiome; 3) mechanisms of HMO utilization by *Bifidobacterium*; 4) perinatal antibiotics and resistance genes in the neonatal gut; 5) gut microbes in neonatal disorders including NEC and sepsis, postnatal growth failure, neonatal cholestasis and jaundice, encephalopathy of prematurity, and predisposition to noncommunicable diseases; 6) debating probiotics use in the NICU; 7) translating neonatal microbiome science into improved health outcomes; 8) therapeutic interventions with prebiotics and probiotics. Created with BioRender.com.

The goal of this special focus issue was to assess the current state of neonatal microbiome science. This is accomplished through 32 articles that deeply explore eight research themes (Figure 1). The areas covered include basic concepts in neonatal gut microbial colonization and functional maturation; dietary influences on the neonatal gut microbiome; mechanisms of human milk oligosaccharide (HMO) utilization by *Bifidobacterium*; perinatal antibiotics and resistance genes in the neonatal gut; gut microbes in neonatal disorders including necrotizing enterocolitis (NEC) and sepsis, postnatal growth failure, neonatal cholestasis and jaundice, encephalopathy of prematurity, and predisposition to noncommunicable diseases; debating probiotics use in the neonatal intensive care unit (NICU); translating neonatal microbiome science into improved health outcomes; and therapeutic interventions with prebiotics and probiotics.

## Part 1: basic concepts in neonatal gut microbial colonization and functional maturation

The special focus issue opens with an overview of factors that influence the development of the gut microbiota and their resulting small molecules (metabolites) early in life by Henrik Roager et al.^3^ Microbial colonization is influenced by delivery mode, gestational age, host genetics, feeding regimen, geography, perinatal antibiotic exposure, and malnutrition. These factors subsequently influence the transformation of carbohydrates, proteins, lipids, and bile acids into short-chain, branched-chain, and aromatic fatty acids and gases by gut microbes. Mechanisms by which microbial metabolites impact the composition and function of the wider gut ecosystem are explored; these include cross-feeding networks and syntrophy (obligately mutualistic metabolism), competitive interactions, and direct antimicrobial activity. The review concludes with a discussion of mechanisms by which microbial metabolites mediate host-microbial cross-talk in early life via host cell receptors to influence intestinal barrier function, gastrointestinal motility, and metabolic health.^3^

This overview is followed by a pair of research papers that provide important new granularity into the development and maturation of the newborn metabolome and metagenome. Ouyang and associates of Zhigang Ren, Xinyu Liu, and Petia Kovatcheva-Datchary performed both liquid chromatography-mass spectrometry-based metabolomic analysis and microbiota sequencing on stool sampled longitudinally from 152 Chinese newborns throughout the first year of life.^4^ The most abundant microbial metabolic pathway in the neonatal gut is lipid metabolism, primarily acylcarnitines and bile acids. Developmental trajectories of the gut metabolome are particularly impacted by cesarean versus vaginal delivery and by early weaning versus sustained breastfeeding. In the second research paper, Minot and colleagues from the laboratory of Jim Kublin conducted a gene-level microbiome analysis with stool obtained from a longitudinal mother-infant birth cohort and augmented their data with publicly available infant metagenomic data sets.^5^ Strain-level de novo analysis of 4,441 metagenomes from 1,162 infants revealed groups of organisms that colonize the newborn gut according to patterns of correlated abundance. Previously unidentified early-life microbial consortia are described, including one group of species spanning *Bacteroides*, *Paraprevotella*, and *Phocaeicola* whose presence is strongly correlated with positive growth in a cohort of infants with cystic fibrosis.^5^

To conclude the first section, Chapman and Stewart discuss key challenges that must be addressed to maximize the impact of future studies of the neonatal gut microbiome.^6^ First, given the large number of covariates and confounders that impact the neonatal gut microbiome, representative cohorts must be recruited into well-designed studies. Multi-center randomized controlled trials (RCTs) maximize the size and diversity of the cohort and may provide initial insights into potential mechanisms. Incorporating methods including restriction, matching, stratification, and multivariate analysis into study designs can control for confounders. Second, given that accurate analysis of the neonatal gut microbiome is dependent upon accurate detection of microbiota composition, the design and selection of methods for the processing of stool samples (sample collection, storage, microbial DNA extraction, or culture) must be carefully considered to minimize the potential for bias. Third, to translate descriptive studies of the neonatal gut microbiome into therapeutic opportunities, mechanisms of interaction must be identified between the gut microbiota and the neonatal host. Among the current menu of tools are rodent and pig models for the preterm and term neonatal gut, organoid models, and *in vitro* co-culture systems. This roadmap of best practices for overcoming existing challenges could improve the quality of future neonatal gut microbiome research.^6^

## Part 2: dietary influences on the neonatal gut microbiome

The second part of this special focus issue considers the influences of the neonatal diet and specific dietary components on the developing gut microbiome. To open this section, Ames, Lotoski, and Azad comprehensively review how differences in neonatal nutritional sources – breastfeeding, expressed mother’s milk fed from a bottle, donor human milk, or formula – shape gut microbial communities, infant immune development, and health outcomes.^7^ Although each of these feeding sources contains the essential macro- and micronutrients to support newborn growth, only mother’s own milk changes over time as the infant ages and contains the complete array of immune cells, immunoglobulins, signaling proteins, bioactive enzymes, microbes, and fatty acids that are variably present in donor human milk and mostly absent from formula. Most oligosaccharides are unaffected by pasteurization and freezing, although the fructooligosaccharides and galactooligosaccharides commonly used as prebiotics in infant formula are structurally and functionally distinct from the HMOs that are highly abundant in human milk. The review concludes by exploring the mechanisms by which diet and gut microbes shape early-life immune system maturation. Critical events include the weaning reaction that is induced by the introduction of complementary foods and the early-life epigenetic modifications that regulate gene expression and developmental programming of the infant immune system.^7^

The next article, written by Dinleyici and associates of Yvan Vandenplas, systematically reviews functional effects of HMOs in the newborn intestine.^8^ HMOs are a structurally diverse class of non-digestible glycans, with the three most abundant categories in human milk being neutral fucosylated HMOs, acidic sialylated HMOs, and neutral non-fucosylated HMOs. HMO concentrations and profiles differ between mothers and are affected by many variables including genetics, environment, and maternal diet; the most important determinants are maternal secretor status and Lewis blood-type, which directly affect HMO fucosylation. HMOs act in tandem with other bioactive components to influence numerous biological processes. Although total HMO intake is not related to infant growth or adiposity, specific HMO-microbe pairs potentially affect anthropometry given that increased fucosylated HMOs positively correlate with body composition. Bacterial fermentation of HMOs produces short-chain fatty acids (SCFAs) and lowers intestinal pH, promotes cross-feeding networks to stabilize communities of beneficial bacteria, and promotes maturation of the immune system and gut barrier. HMOs are linked to decreased risks of NEC, intestinal and systemic infections, allergic and atopic disorders, and neurocognitive delays. However, whether HMO supplementation of infant formula improves clinical outcomes remains a topic of debate given the limited available evidence to date.^8^

These two topic overviews are followed by a research paper from David Barnett and colleagues in which stool microbiota was profiled from 1,023 one-month-old infants and combined with a quantitative high-performance liquid chromatography-mass spectrometry analysis of 15 abundant HMOs in milk from the infants’ mothers.^9^ Gut microbial community diversity is decreased by both exclusive breast feeding and postnatal antibiotic exposure, but not by maternal exposure to antibiotics during pregnancy or neonatal exposure to oral antifungals. Natural variation in the HMOs consists of four distinct groups corresponding to maternal secretor and Lewis blood-type status that influence infant gut microbiota composition. In a subset of exclusively formula-fed infants, galactooligosaccharide supplementation was shown to impact gut microbial community composition. The authors conclude that natural variation in HMO content has the potential to influence the gut microbiota of breastfed infants.^9^

## Part 3: mechanisms of human milk oligosaccharide utilization by *Bifidobacterium*

The next section of the special focus issue delves deeply into the basic mechanisms by which specific bifidobacterial strains metabolize HMOs into bioactive components. The section opens with a review by Yang and colleagues of Xiangchen Meng, which describes interactions between HMOs and the dominant subspecies of *Bifidobacterium longum* in the infant gut.^10^ *B. longum* is vertically transmitted at birth and enhances its own colonization through the extracellular secretion of adhesins, extracellular vesicles, and exopolysaccharide. Complete metabolism of HMOs requires specific transporters, catabolic enzymes including glycoside hydrolases, and transcriptional regulators. Strains within the *B. longum* species possess a large and varied array of this enzymatic machinery, and specific pathways of HMO degradation and subsequent cross-feeding networks are described for *B. longum* subsp. *infantis* (*B. infantis*) and *B. longum* subsp. *longum*. Beneficial effects of HMOs and *B. longum* may be mediated by a variety of mechanisms that include producing SCFAs, aromatic lactic acids, and other beneficial metabolites; shaping the resident gut microbial communities; increasing goblet cell numbers and secretion of intestinal mucus; increasing the expression of junction proteins within the intestinal barrier; and supporting the maturation of gut-associated lymphatic tissue and an anti-inflammatory intestinal environment. The review concludes by taking inventory of commercially available combinations of prebiotics and probiotics and highlights limitations to the existing clinical evidence supporting the use of *B. longum* and HMOs as synbiotics.^10^

The next two articles, complementary research papers from the David Sela laboratory, provide important new insights into mechanisms of substrate utilization by *B. infantis*.^11,12^ HMOs are studied primarily as a prebiotic carbon source. However, they also contain nitrogen within *N*-acetylglucosamine (NAG) residues. Li et al. aimed to test the hypothesis that *B. infantis* harnesses nitrogen from NAG-containing HMOs. Indeed, *B. infantis* grows on HMOs as a primary nitrogen source and incorporates NAG nitrogen into its proteome in a strain-specific manner. The efficiency by which *B. infantis* metabolizes nitrogen is inversely proportional to intracellular levels of 2-oxoglutarate, a marker of nitrogen unavailability. Comparative transcriptomic and proteomic profiles further support global cellular changes resulting from nitrogen metabolism by *B. infantis*. This study advances our understanding of *B. infantis* HMO utilization phenotypes and of gut microbial nitrogen metabolism.^11^ The second article focuses on another non-digestible component of human milk, urea, which is present in high concentrations and typically is excreted as metabolic waste. You et al. tested the hypothesis that commensal *B. infantis* utilizes urea as a nitrogen source to benefit the infant by providing reclaimed nitrogen within the microbiome. The authors show that *B. infantis* thrives on urea as a primary nitrogen source by activating a chromosomally encoded urease gene cluster that catalyzes the rate-limiting hydrolytic step to make ammonia available for downstream anabolism. These data are further supported by the broad distribution of isotopically labeled urea nitrogen throughout the *B. infantis* proteome, and by the induction of pathways specifically involving branched chain and other essential amino acids that may be beneficial to the neonate.^12^

## Part 4: perinatal antibiotics and resistance genes in the neonatal gut

In high-income countries, antibiotics are administered to 35% of pregnant women^13^ and to 70% of infants.^14^ In low- and middle-income countries antibiotic exposure is estimated to be five times higher.^15^ Multiple international guidelines, including those issued by the World Health Organization,^16^ recommend routine antibiotic administration just prior to cesarean delivery to reduce the risk of maternal post-operative infections. However, this practice exposes the newborn to antibiotics during delivery with potentially unintended consequences. This section of the special focus issue explores the effects of intrapartum antibiotic use on gut microbial communities and infant health.

Two primary research papers add to our understanding of how intrapartum antibiotics affect the neonatal gut microbiome and newborn health. Jokela and colleagues in the laboratory of Anne Salonen performed quantitative fecal microbiota profiling among 92 healthy Finnish infants during the first year of life.^17^ Infants were born vaginally with or without intrapartum antibiotics, or they were born via Cesarean delivery with intrapartum antibiotics. Five fecal samples were collected from each infant longitudinally, and symptoms of gastrointestinal function and discomfort were recorded as indicators of infant well-being. The authors reveal that patterns of colonization of the newborn gut following vaginal delivery are dramatically altered both by Cesarean delivery and by intrapartum antibiotics; among these alterations is decreased absolute abundance of *Bifidobacterium*. Mediation analysis revealed that gut microbiota alterations attributed to Cesarean delivery or intrapartum antibiotics lead to increased crying intensity, abdominal pain, and rate of defecation.^17^ In another study, Bossung and collaborators of Christoph Härtel analyzed longitudinal gut microbiota signatures from infants born to 40 women undergoing elective Cesarean sections who were randomized into two groups in an exploratory study.^18^ Mothers in the intervention group received a single dose of intravenous cefuroxime after umbilical cord clamping to prevent antibiotic exposure to the newborn, while the control group received intravenous cefuroxime 30 min before skin incision, exposing the newborn to cefuroxime that was detected in cord blood. In the control group, meconium microbiota were modified by cefuroxime in a dose-dependent manner, and at 1 y of life differences persisted in fecal microbiota profiles between the two groups, indicating that the timing of perinatal antimicrobial prophylaxis could have long-term implications for the infant microbiome.^18^

This section concludes with a review by Samarra and coauthors from the laboratory of Maria Carmen Collado that outlines the current state of knowledge of vertical transfer of antibiotic resistance genes (ARGs).^19^ The authors describe how the mother-infant resistome, defined as the set of ARGs present in a population of bacteria, is determined by gut microbiota composition given that the majority of ARGs are carried by a select number of taxa. Increased presence of mobile genetic elements including plasmids, integrons, bacteriophages, and transposable elements may facilitate mother-infant transfer of resistance to beta-lactamases, tetracyclines, macrolides, aminoglycosides, and quinolones. Mechanisms of intrinsic, acquired, and adaptive antibiotic resistance in the context of vertical transmission are described; the influence of gestational age, delivery mode, feeding strategy, and perinatal antibiotic exposure are discussed; and strategies to reduce antibiotic resistance in mother-infant dyads via diet or supplementation with prebiotics or probiotics are proposed.^19^

## Part 5: neonatal gut microbes in disease

The next section in this special focus issue describes characteristic changes in gut microbiome signatures in the context of highly prevalent conditions associated with prematurity.

### Necrotizing enterocolitis and sepsis

NEC is an inflammatory intestinal condition that affects 7% of newborns with very low birth weight (VLBW, <1,500 g). Ischemic necrosis of the bowel can allow microbes to translocate from the gut lumen into the bloodstream resulting in sepsis and occasionally multiorgan failure. Mortality rates from NEC approximate 20–30%, and survivors may be plagued with short bowel syndrome, parenteral nutrition-associated liver injury, and long-term neurodevelopmental delays. However, not all cases of NEC result in sepsis; rather, some cases of NEC are preceded by sepsis. Despite decades of research, the etiologies of NEC and sepsis in preterm neonates remain incompletely understood.^20,21^

Duess and coauthors from the laboratory of David Hackam review the evidence implicating abnormal microbial signaling through intestinal Toll-like receptor 4 (TLR4) in gut barrier injury. Epithelial cell death can occur by apoptosis, autophagy, and necroptosis. There may be impairment of immune function, intestinal epithelial cell proliferation, and mucosal healing.^22^ Compared to the full-term infant gut, the preterm infant gut expresses TLR4 at higher levels and harbors an increased abundance of gram-negative Proteobacteria whose cell walls are enriched with the TLR4 ligand lipopolysaccharide (LPS). TLR4 activation stimulates a robust nuclear factor κB-dependent inflammatory response, induces loss of enteric glia leading to intestinal dysmotility, and decreases endothelial nitric oxide synthase resulting in mesenteric vasoconstriction and intestinal ischemia. Current treatments rely on antibiotics, cessation of enteral feeds, and surgical resection of severely necrotic bowel. However, given the emerging evidence that specific gut microbial community changes influence susceptibility and outcomes in NEC and sepsis, and new preclinical evidence that maternal microbial metabolites targeting aryl hydrocarbon receptors ligands may confer protection by dampening TLR4 signaling in the neonatal gut, more focused microbiome-targeting strategies could be on the horizon.^22^

Adding to our knowledge of microbial susceptibility factors for sepsis, Mukhopadhyay and colleagues in the laboratory of Kyle Bittinger tested the hypothesis that VLBW infants who were considered to be at low risk of early-onset sepsis could be identified based on distinct microbiome composition including reduced early bacterial acquisition.^23^ Low-risk status was based on meeting four clinical criteria: birth by Cesarean delivery, absence of spontaneous or induced labor, rupture of membranes not prior to delivery, and preterm delivery due to noninfectious medical indications. They compared microbiome signatures from low-risk newborns who were not administered routine antibiotics for sepsis prevention in the first 24 h of life to those from standard-risk preterm neonates who received antibiotics. Within the first week of life, stool from low-risk newborns contains lower bacterial load, lower *Escherichia coli*, and higher *Staphylococcus epidermidis*. Over time, differences emerge in the abundance of antibiotic resistance genes and virulence factors. Nine infants in this study developed late-onset bacteremia; eight of these cases occurred in standard-risk newborns who had received antibiotics in the first 24 h of life. Blood cultures grew bacteria that were present prior to the initiation of antibiotics, but these bacteria were also identified in infants who did not develop bacteremia. These findings raise the possibility that specific microbiome features and clinical criteria could ultimately identify which VLBW neonates are at highest risk of sepsis and could decrease unnecessary antibiotic exposure to low-risk neonates.^23^

### Postnatal growth failure

In the next article, Neves and coauthors Amy Hair and Geoffrey Preidis explore the role of the gut microbiome in neonatal growth failure, which affects half of all preterm VLBW infants.^24^ They review the preclinical models that reveal causal links between gut microbial community alterations and postnatal growth. Microbial fermentation products including SCFAs, lactate, and indole-3-lactic acid can improve growth by regulating energy extraction from milk, bone and somatic growth, gut barrier function, pathogen colonization, and inflammation. To determine whether any human gut microbiome features consistently associate with neonatal growth failure, the authors performed a systematic review of the literature. Fourteen relevant articles were identified, all using 16S sequencing, but there was little consensus among studies regarding specific taxa that correlate either positively or negatively with growth. Importantly, no two studies used the same definitions of growth (e.g., infant weight, length, or head circumference reported as absolute value, percentile, rate of change, or z-score and infant age reported as postnatal age or postmenstrual age). This led them to hypothesize that different definitions of neonatal growth will influence which taxa correlate with growth. To test this hypothesis, they performed an exploratory secondary analysis of 245 preterm infant stools and identified associations between 16 clinically relevant definitions of neonatal growth and gut microbial signatures. Indeed, every definition of growth was correlated with a different set of microbial taxa. The authors recommend that future studies pre-register all protocols and reveal all correlations tested to provide greater transparency regarding the potential risk of type I error. These guidelines could accelerate the development of microbiota-targeting therapies that prevent growth failure among premature neonates.^24^

### Neonatal cholestasis and jaundice

Physiologic cholestasis, defined as impaired bile flow from the liver to the intestine, occurs in 20% of preterm births and is associated with poor outcomes including growth failure.^25^ Despite limited efficacy data, ursodeoxycholic acid (UDCA) is commonly prescribed to stimulate bile flow. Although UDCA is generally well tolerated, little is known regarding how the drug interacts with gut microbes and other bile acids in the extremely preterm neonatal gut. Lynch and colleagues in the laboratory of Geoffrey Preidis conducted a longitudinal nested case–control study of 24 extremely preterm, extremely low birth weight infants, half of whom developed cholestasis.^26^ Acquisition of bile salt hydrolase (BSH) genes carried by *Clostridium perfringens* is the hallmark feature of microbiome development in non-cholestatic infants. This developmental pattern is delayed in cholestatic neonates. Quantitative bile acid metabolomics and a novel *in vitro* BSH enzyme activity assay were used to confirm that cholestatic infants lack many of the microbial-derived unconjugated bile acids. Interestingly, in cholestatic infants treated with UDCA, total fecal bile acid quantities are restored to normal levels; however, almost all of these fecal bile acids are UDCA, suggesting that the administered UDCA passes through the intestine unabsorbed. The majority of bile acids in extremely preterm newborns are atypical bile acid isomers that are present in fetal development. Fetal isomeric bile acids and BSH activity are positively associated with infant growth. Thus, delayed acquisition of BSH and delayed production of microbial-derived unconjugated bile acids could help identify extremely preterm neonates who are at high risk of cholestasis and growth failure.^26^

Not all neonatal jaundice is caused by cholestasis. Another prevalent disorder of prematurity is unconjugated or indirect hyperbilirubinemia, which in severe cases can lead to encephalopathy, brain damage, and death if not promptly treated with phototherapy or exchange transfusion. Chen and associates working with Wenhao Zhou, Shujuan Zeng, and Mingbang Wang analyzed fecal microbiota and metabolome profiles from 98 neonates with jaundice and 98 healthy controls.^27^ A combination of machine learning, causal inference, and clinical prediction models revealed that specific gut bacteria affect the risk of hyperbilirubinemia by altering intestinal bile acid metabolism. In the future, focused panels of gut microbes and bile acids could potentially identify neonates who are at highest risk of severe hyperbilirubinemia, prompting early intervention before serum bilirubin rises to dangerous levels.

### Encephalopathy of prematurity

Brain injury is common among infants born prematurely, with up to 10% experiencing significant motor deficits including cerebral palsy, and 25–50% experiencing some form of neurocognitive disability.^28^ Encephalopathy of prematurity primarily consists of periventricular leukomalacia, a distinctive form of cerebral white matter injury that is accompanied by neuronal/axonal disease. Neonatal brain injury is caused by numerous factors that disrupt the microbiota-gut-immune-brain axis.^29,30^

To elucidate gut microbiome changes that occur with perinatal white matter injury, a team led by David Seki and David Berry combined fecal metagenomic sequencing with routine cranial magnetic resonance imaging performed at term-equivalent age among 30 extremely preterm infants with ELBW.^31^ Brain injuries, which included periventricular hemorrhagic infarction, intraventricular hemorrhage, cerebellar hemorrhage, and periventricular leukomalacia, were associated with reduced brain volume and enlarged subarachnoid space. Metagenomic sequencing was performed on 50 fecal samples from 24 infants without severe brain injury and 13 samples from 6 infants with severe brain injury. Loss of genetic functional redundancy and enrichment in iron scavenging and nitrate reduction pathways are key metagenomic traits that are associated with the proliferation of potential pathobionts that may be involved in further aggravating severe brain damage. This study highlights microbial metabolic changes that could be targeted to protect against encephalopathy of prematurity.^31^

### Predisposition to noncommunicable diseases

The final review of this section, written by Alessio Fasano and Roberto Berni Canani and colleagues, explores factors that shape a newborn’s gut microbial communities to modify the risk of obesity, diabetes, cardiovascular diseases, malnutrition, allergies, celiac disease, and inflammatory bowel diseases across different life stages.^32^ A bidirectional relationship exists between maternal microbiota and pregnancy complications including gestational diabetes mellitus and preterm birth that may provide opportunities to predict and prevent these complications and improve fetal health and pregnancy outcomes. Given the importance of human milk composition and its repercussions on microbiome engraftment and development, along with the current lack of clarity regarding how specific components of human milk impact long-term outcomes, opportunities abound to identify microbial markers and interventions to improve maternal-neonatal microbiota care. In the first year of life, altered microbial communities are linked to changes in epigenetics, gut barrier permeability, and immune responses that predispose to microbiota-driven chronic inflammatory conditions. Strategies for attenuating the pathogenic effects of gut microbiota in non-communicable diseases are discussed, including interventions that target the maternal diet, human milk composition, and neonatal gut microbial communities via human milk, infant formula, complementary feeding, and biotic approaches. This perspective transforms diseases from noncommunicable to communicable and highlights how breakdown of the symbiotic relationship between the gut microbiota and its host could play a causal role.^32^

## Part 6: debating probiotics use in the neonatal intensive care unit

One of the most hotly debated topics in neonatology today is the question of whether probiotics should be given routinely to preterm neonates in the NICU. Clinical trials have tested probiotics in the NICU for more than four decades, and the total body of evidence is substantial.^33^ One recent Cochrane review^34^ and four network meta-analyses, each including more than 10,000 preterm infants,^35–38^ suggest that probiotics may reduce the risk of NEC by as much as 50% and identify specific probiotic strains and combinations that are associated with the greatest benefit.

Despite this evidence, many providers are reluctant to administer probiotics in the NICU setting.^39^ The most common concerns relate to safety. The lack of pharmaceutical-grade probiotic products of sufficient microbiological quality to meet the specific needs of vulnerable preterm neonates was made apparent in 2014 when a fatal case of gastrointestinal mucormycosis in a preterm infant was traced to a contaminated probiotic product.^40,41^ Although multidisciplinary strategies to minimize product contamination are proposed,^42^ by their very nature live microorganisms have the potential to translocate from the gastrointestinal tract and cause harm to immunologically naïve neonates. Several case reports have documented invasive infections attributed to probiotic products that were not attributed to contamination,^43^ and the recent death of a preterm infant with ELBW who had received a probiotic supplement prompted the US Food and Drug Administration in September 2023 to warn providers against administering probiotics to preterm infants.^44^ The American Academy of Pediatrics does not endorse routine probiotics use in the NICU,^45^ whereas both the American Gastroenterological Association^46^ and the European Society for Pediatric Gastroenterology Hepatology and Nutrition^47^ conditionally recommend the use of certain probiotics in the NICU. A conditional recommendation indicates that guideline panels concluded that the desirable effects of administering probiotics probably outweigh the undesirable effects, but they were not confident enough to issue a strong recommendation. Conditional recommendations also suggest that most caregivers would offer probiotics to preterm neonates, but clinicians should recognize that different choices will be appropriate for different families and that management decisions should be made that are consistent with the family’s values and preferences.^39^ The next section of the special focus issue adds to the ongoing debate by presenting two perspectives on the potential role of probiotics in the NICU.

Mercer and Arrieta review our current understanding of how probiotics modify the preterm neonatal gut microbiome, mechanisms by which probiotics promote neonatal health, and clinical considerations for the use of probiotics in this vulnerable population.^48^ The predictable patterns of colonization, succession, and establishment of stable communities observed in healthy full-term newborns are altered in preterm infants due to numerous factors including immature gut and immune physiology, antibiotics and other medications, colonization by hospital-acquired microbes endemic to the NICU environment, and various parenteral and enteral feeding strategies. A comprehensive review of the literature summarizes supplementation strategies with *Bifidobacterium* spp., *Lactobacillaceae*, *Saccharomyces* spp., and multi-genus probiotic formulations that influence infant gut microbial community composition, diversity, and functional metagenomic potential. These therapies can shape the infant metabolome and immune system to more closely reflect that of the healthy full-term infant. Although accumulating evidence from numerous RCTs suggests potential benefits of probiotics in terms of reduced risk of NEC, sepsis, and neonatal mortality, key questions remain regarding safety in vulnerable subpopulations, the timing of initiation, the frequency and duration of administration, and differing outcomes among probiotic formulations. Thoughtful recommendations are made for determining the best practices for probiotic use in premature infants.^48^

In the second review in this section, DeVeaux and associates of Gautam Dantas, Barbara Warner, and Phillip Tarr highlight major limitations in the existing evidence base that make it difficult to support routine probiotics use in the NICU.^49^ Three classes of microbiota-targeting therapies are comprehensively reviewed: probiotics, prebiotics, and synbiotics. Compared to probiotics, prebiotics and synbiotics have been studied in considerably fewer RCTs. Prebiotics appear to be safe and well tolerated by preterm infants, but their benefits are likely modest at best. Synbiotics may offer synergy between probiotics and prebiotics, but additional RCTs that include larger numbers of preterm infants are needed to rigorously determine safety and efficacy in this population. The large body of existing evidence examining single- and multiple-strain probiotics on neonatal outcomes (NEC, late-onset sepsis, feeding intolerance, and all-cause mortality) contains striking heterogeneity with respect to study design and probiotic strain, dose, duration, and viability. An independent assessment of a subset of the RCTs included in the meta-analyses that have informed societies’ clinical practice guidelines revealed substantial risk of bias and inconsistent results among the individual trials. Importantly, most RCTs did not meet the rigorous criteria for trials that would qualify probiotic interventions as drugs. This perspective highlights potential limitations of meta-analyses that combine the results of heterogeneous studies into single-point estimates for a group of therapies.^49^

## Part 7: translating neonatal microbiome science into improved health outcomes

Given that key questions remain regarding the use of current probiotic formulations in the neonatal period, Part 7 of this special focus issue considers future microbiota-targeting interventions that aim to leverage knowledge of the neonatal gut microbiome to develop safe and effective therapies. This section opens with a review by Mills and colleagues of Paul Ross that considers interventions based on the most abundant bacterial species of the neonatal gut, *B. longum*.^50^ Four subspecies of *B. longum* confer metabolic activities including production of SCFAs, indole-3-lactic acid, capsular polysaccharides and exopolysaccharides, serine protease inhibitors, and conjugated linoleic acid that collectively shape the host gastrointestinal niche and enhance bifidobacterial colonization. A comprehensive review of 64 published RCTs (44 trials investigating *B. longum* alone, 20 testing *B. longum* in combination with other microbial strains or prebiotics) reveals that *B. longum* might decrease risks of childhood disorders including NEC, sepsis, gastroschisis, diarrhea, irritable bowel syndrome, inflammatory bowel disease, developmental disorders, autism spectrum disorders, allergic disorders, and atopic dermatitis. *B. longum* supplementation also might decrease risks of a variety of disorders in adults and the elderly. The authors recommend continued research into the beneficial properties of *B. longum* given that this species could significantly improve aspects of human health from birth throughout the lifespan.^50^

In the next review in this series, Katri Korpela and Willem de Vos discuss general principles and strategies by which disruptions in the newborn gut microbiota may be restored.^51^ They make the case that natural newborn gut colonization and development is becoming rare; in many regions more than half of infants are exposed at birth to interventions that disrupt natural microbiota colonization. Although the consequences of these impacts on public health are not fully understood, there is evidence that increased abundance of pathobionts and decreased abundance of beneficial taxa may induce inflammation, weaken the gut barrier, subject the immune system to heightened sensitivity, and predispose the host to obesity and chronic immune-mediated diseases. The current evidence and future potential of microbiota-targeting strategies for full-term infants is reviewed, with a focus on probiotics, vaginal seeding, and maternal fecal microbiota transfer (FMT). Among the most compelling evidence to date, supplementation with a four-strain probiotic and fructooligosaccharide product may reduce the risk of IgE-associated allergy in infants delivered by Cesarean section. Although only a few small pilot studies have been conducted with vaginal seeding and FMT, the authors analyzed available microbiota sequencing data to illustrate how FMT minimizes the microbiota differences between Cesarean-delivered and vaginally delivered infants more completely than strategies based on probiotics or vaginal seeding. These intriguing findings are motivating ongoing investigations of vaginal seeding and maternal FMT and their impacts on infant gut microbiota development and immune health.^51^

Next, Liu and colleagues of Ri-hua Xie and Yan He critically review the published trials that examine vaginal seeding and FMT as means of addressing gut microbiota alterations in infants born by Cesarean delivery.^52^ The global incidence of Cesarean delivery exceeds 21% of live births and has been associated with increased risks of neurological disorders, chronic immune diseases, obesity, allergic diseases, and type 1 diabetes mellitus. Vaginal microbiota exposure provides the infant with microbes not typically encountered in a sterile Cesarean delivery. Several study designs were identified. Four studies (421 mother-infant pairs) tested maternal vaginal swabs applied to the baby’s mouth, face, and body. One study (47 mother-infant pairs) assessed the impact of processed maternal vaginal solution administered directly into the baby’s mouth. One study (214 mother-infant pairs) tested pre-processed maternal fecal material added to the infant’s first human milk feed. In general, these treatments shift newborn gut microbiota profiles to become more similar to those of full-term vaginally delivered infants. Although safety data from these small pilot studies are reassuring, significant controversy exists regarding the possibility that fecal and vaginal samples could contain pathogens; furthermore, variations in operational procedures make it difficult to assimilate data across studies. Further studies are warranted given that the mechanisms underlying the effects of these interventions, including clinically relevant outcomes, remain unclear.^52^

These reviews are followed by two original research papers that investigate potential future microbiota-targeting therapies in animal models of neonatal disorders. The first builds upon prior studies in which fecal filtrate transfer (FFT) – whereby donor fecal matter is processed to remove bacteria, fungi, and parasites but still contains phages, eukaryotic viruses, extracellular vesicles, and bacterial metabolites as a potentially safer alternative to FMT – prevents NEC in susceptible piglets.^53^ To test the hypothesis that the virome is a crucial protective component of FFT, Offerson et al. in the laboratory of Anders Brunse separated FFT into two fractions (isolated virus-like particles and virus-depleted filtrate residual fluid), then compared these two fractions to native FFT in preterm Cesarean-delivered piglets.^54^ Transfer of the virome, but not the virus-depleted fraction, is as effective as native FFT in reducing the severity of NEC-like pathology. Virome transfer reduces the relative abundance of NEC-associated pathogens including *Klebsiella pneumoniae* and *C. perfringens* but also is associated with early-onset diarrhea.^54^ In the second study, Lu et al. in the laboratory of Erika Claud tested a probiotic intervention in pregnant mice subjected to LPS-induced late-gestation inflammation that mimics chorioamnionitis.^55^ Maternal immune activation interferes with the development of the blood–brain barrier (BBB), placing offspring at risk for adverse neurodevelopmental outcomes. The authors hypothesized that daily gavages with probiotic *Limosilactobacillus reuteri* ATCC PTA 6475 to lactating dams during the first 3 weeks of postnatal life would alleviate adverse effects of maternal immune activation on offspring BBB development and function. They show that late-pregnancy maternal inflammation, induced by one injection of intraperitoneal LPS on gestational day 16, increases offspring BBB permeability and impairs BBB development, evidenced by reduced total blood vessel volume on magnetic resonance imaging and distinct shifts in the serum and brain metabolomes. Probiotic treatment normalizes BBB developmental and functional deficits, promotes entry of specific bacterial metabolites into the brain, and improves offspring spatial learning at 12 weeks of age as determined on the Morris water maze.^55^

The next article in this translational research section is a narrative review by David Mills and colleagues that describes the series of basic and translational discoveries that facilitated the commercial production of HMO-consuming bifidobacteria for premature, human milk-fed neonates.^56^ In the early 1900s, bifidobacteria were first identified and were associated with fecal acidification and reduced risk of infant gut-borne pathogens. Over the next several decades, HMOs were isolated, purified, and characterized. Various consumption mechanisms among bifidobacteria, and their resulting fermentation products, were elucidated. Fermentation products include acetic and lactic acids, indole lactic acid, and a variety of aromatic acids, the health benefits of which are now emerging from animal models and human trials. As high-throughput sequencing improved our understanding of the genomic diversity and the geographic differences of HMO-consuming bifidobacteria in the human gut, an argument can be made that their underrepresentation in some regions of the world is an unintended consequence of industrialization and modern lifestyles. An overt mechanistic link is presented between select supplemented probiotics and improved infant health. Functional synbiotic applications may facilitate discoveries and translations that advance human health in numerous settings.^56^

This section concludes with a research paper that illustrates how population-wide studies of gut microbiome development can be altered by global events. Zhang and colleagues of Hein Tun, Francis Chan, and Siew Ng revealed how gut microbiome maturation in the first year of life was altered during the COVID-19 pandemic.^57^ Shotgun metagenomic sequencing was performed on a total of 521 stool samples from 134 infants and 103 stool samples from mothers in the Greater Bay Area of China. These samples were divided into two longitudinal cohorts based on whether they were collected before or during the pandemic. The early-life gut microbiome developmental trajectory was substantially altered during the pandemic, evidenced by decreased microbial community richness and diversity and altered relative abundance of multiple species. Interestingly, samples collected during the pandemic exhibited decreased richness and diversity of antimicrobial peptide resistance genes, suggesting compromised gut microbiome adaptability, and decreased richness and diversity of ARGs, which could reflect the decreased hospital visits and decreased antibiotic use amidst social isolation. Whether these alterations will negatively impact infant health, or whether increased hygiene and sanitation predispose to the development of atopic, metabolic, and inflammatory diseases in general, are key questions that remain under investigation.^57^

## Part 8: therapeutic interventions with prebiotics and probiotics

The final section of this special focus issue highlights prebiotic and probiotic interventions that modulate the newborn gut microbiota. The first article features a three-arm RCT in which Raakel Luoto and colleagues compared the effects of *Lacticaseibacillus rhamnosus* GG ATCC 53103 (1 × 10^9^ cfu/d for the first month, 2 × 10^9^ cfu/d for the second month) or a 1:1 mixture of galactooligosaccharide and polydextrose (600 mg/d for the first month, 1200 mg/d for the second month) versus placebo on the longitudinal gut microbiome development of 43 moderate- and late-preterm infants (born after 32–36 weeks of gestation) in comparison to 9 healthy full-term infants.^58^ Preterm newborns have decreased gut microbial community richness and diversity that normalizes by 1 month of age, although taxonomic differences persist until 6 months. Neither the prebiotic nor the probiotic intervention affected alpha diversity, although both treatments made the preterm gut microbiome more similar to that of healthy full-term infants.^58^ The next article in this section by Wang and associates of Yanjie Deng, Jingyu Yan, and Ming Li begins with an elegant set of preclinical studies that reveal how the combination of sialic acid, a key component of sialylated HMOs, and three probiotic strains (*Bifidobacterium breve* M-16 V, *L. rhamnosus* HN001, and *Limosilactobacillus fermentum* CECT5716) gavaged to lactating rats promotes gut microbiota maturation and attenuates severe allergic responses in the neonatal rat pups via increased production of 6’-sialyllactose in mother’s milk.^59^ Transcriptome studies of the rat mammary gland and complementary experiments in human mammary epithelial cells identified a potential pathway by which SCFAs induce 6’-sialyllactose synthesis in the mammary gland by signaling through both G protein-coupled receptor and nuclear receptor pathways. Finally, 66 mothers in Northern China were randomized to receive either placebo or 300 mg sialic acid with 1.5 × 10^10^ total cfu of the probiotic strains daily for 4 weeks starting in the second week of lactation. Milk from mothers receiving the treatment contained higher concentrations of sialylated HMOs, and this correlated with increased infant gut microbial community diversity and increased abundance of *Bifidobacterium* spp. Infants whose mothers received prebiotics and probiotics also had fewer skin rashes and less eczema.^59^

In the final article of this special focus issue, Panigrahi makes a compelling argument for why microbiome science and clinical trials performed in western settings cannot necessarily be extrapolated to low- and middle-income country settings.^60^ He outlines the process by which a synbiotic preparation was selected for a randomized, double-blind, placebo-controlled trial enrolling 4,556 rural Indian newborns.^61^ More than 280 candidate probiotic strains were screened for their ability to strongly adhere to cultured Caco-2 cells, thereby blocking the adherence and translocation of *E. coli*, and to reduce NEC-like disease in rabbit ileal loops. Preclinical studies identified *Lactiplantibacillus plantarum* ATCC 202195 as an ideal candidate strain and determined that 4 weeks of oral administration is safe for rabbit pups. Prebiotic fructooligosaccharide was added to improve *L. planterum* colonization. After a small hospital-based study that confirmed colonization of the probiotic strain in infants, the community-based efficacy trial was launched. Compared to placebo, the synbiotic dramatically reduced the primary outcome, combined incidence of sepsis and death (risk ratio 0.60, 95% confidence interval 0.48–0.74).^61^ The review concludes with a discussion of the challenges and lessons learned conducting neonatal microbiome research in settings that may lack basic infrastructure, including electricity and clean water. Above all, Panigrahi highlights the promise and hope that microbiome science may reduce neonatal morbidity and mortality on a global scale.^60^

## Conclusions

Despite the extraordinary number of recent discoveries that have advanced our understanding of microbial communities within the newborn gut, microbiota-targeting therapies are not yet ready for integration into the routine clinical care of the neonate. For this to occur, clear mechanistic links must be established between therapeutic interventions and clearly defined clinical outcomes, and safety in this vulnerable population must be assessed with rigor. The importance of clinically meaningful outcomes cannot be understated. Although it may be appealing to manipulate preterm newborn intestinal microbial communities so that they mimic those of healthy, full-term newborns, the gut microbial functions that support survival, growth, and protection from devastating comorbidities in an infant born 18 weeks prematurely could differ substantially from those typically found in a healthy newborn. While we continue to learn how genetics, maternal diet and health status, degree of prematurity, perinatal events, and feeding strategies affect microbial colonization and succession patterns, we must remain open to the possibility that successful interventions might require individualization at multiple developmental stages from preconception through childhood.

## Abbreviations

ARG: antibiotic resistance genes
BBB: blood-brain barrier
BSH: bile salt hydrolase
CFU: colony-forming units
ELBW: extremely low birth weight
FFT: fecal filtrate transfer
FMT: fecal microbiota transfer
HMO: human milk oligosaccharide
LPS: lipopolysaccharide
NAG: *N*-acetylglucosamine
NEC: necrotizing enterocolitis
NICU: neonatal intensive care unit
RCT: randomized controlled trial
SCFA: short-chain fatty acids
TLR4: Toll-like receptor 4
UDCA: ursodeoxycholic acid
VLBW: very low birth weight
